# Non-Random Sibling Cannibalism in the Marine Gastropod *Crepidula coquimbensis*

**DOI:** 10.1371/journal.pone.0067050

**Published:** 2013-06-21

**Authors:** Antonio Brante, Miriam Fernández, Frédérique Viard

**Affiliations:** 1 Departamento de Ecología, Facultad de Ciencias, Universidad Católica de la Santísima Concepción, Concepción, Chile; 2 Center for Marine Conservation, Estación Costera de Investigaciones Marinas of Las Cruces, Departamento de Ecología, Facultad de Ciencias Biológicas, Pontificia Universidad Católica de Chile, Santiago, Chile; 3 International Associated Laboratory ‘Dispersal and Adaptation in Marine Species’ (Station Biologique de Roscoff and Center for Advanced Studies in Ecology and Biodiversity [CASEB]), Roscoff, France, and Santiago, Chile; 4 UPMC Univ Paris 06, Adaptation & Diversity in the Marine Environment UMR 7144, Station Biologique de Roscoff, 29682 Roscoff, France; 5 CNRS, UMR 7144, Team Diversity & Connectivity in Coastal Marine Landscapes, Station Biologique de Roscoff, 29682 Roscoff, France; VIB & Katholieke Universiteit Leuven, Belgium

## Abstract

Sibling cannibalism is commonly observed in marine species. For instance, intrabrood cannibalism has been documented in marine gastropods with direct development, suggesting a relationship between embryo behavior and the evolution of life history strategies. However, there has been little effort to document the factors driving sibling cannibalism in marine species. The kin selection theory suggests that the level of relatedness plays an important role in cannibalism patterns. We examined *Crepidula coquimbensis*, a marine gastropod that broods and encloses its brooded offspring in capsules. Encapsulated embryos show sibling cannibalism and high levels of intracapsular multiple paternity. Given these features, cannibalistic behavior may be driven by kin-relatedness. To test this hypothesis, we constructed artificial aggregations of embryos to mimic three levels of relatedness: high, medium and low. For each category of aggregation, the cannibalism rate and benefits (i.e. size at hatching of surviving offspring) were estimated. In addition, at the end of embryo development, we performed parentage analyses to determine if cannibalism was associated with the relatedness between cannibal and victim embryos. Our results show that the intensity of sibling cannibalism increased in aggregations characterized by the lowest level of relatedness. There were important benefits of cannibalism in terms of hatching cannibal size. In addition, cannibalism between embryos was not random: the variation in reproductive success between males increased over the course of the experiment and the effective number of fathers decreased. Altogether, these results suggest that polyandry may play an important role in the evolution of sibling cannibalism in *C. coquimbensis* and that kin selection may operate during early embryonic stages in this species.

## Introduction

Sibling cannibalism is a widespread phenomenon observed in nature [1], [2], [3], [4]: cannibal individuals prey on sibs for food and to escape from limiting resources [5], [6], [7]. Cannibal offspring (i.e. the survivors) benefit from cannibalism in terms of growth rate and nutritional requirements, as reported in various taxonomic groups, such as amphibians [6], [8], sharks [9], insects [10], [11], [12], [13], [14] and mollusks [15]. The cannibal victims obviously pay the highest possible cost by being killed. From the parental perspective, sibling cannibalism may be unfavorable, particularly when the overall parental reproductive value is reduced [16].

Various studies have tried to determine the factors promoting sibling cannibalism. In terrestrial species, experimental data show that food limitation acts as an important trigger (e.g. spadefoot toad tadpoles [7], tiger salamander larvae [17], parasitic wasps [18], [19] and a terrestrial gastropod [20]). For example, higher cannibalism rates have been observed in spadefoot toad tadpoles for which there are few heterospecific prey [7]. In aquatic systems, oxygen may also become a limiting factor for embryos in species that aggregate their offspring [21], [22], [23]. For instance, in the marine gastropod *Acanthina monodon*, which encapsulates its eggs during their development, the cannibalism rate has been shown to increase as levels of oxygen availability decrease inside capsules [24].

Less work has been carried out to explore the sib-sib interaction and thus the mechanisms driving the cannibalistic behavior of sibs and the adaptive value of this type of behavior. The kin selection model [25] is an interesting framework for understanding the evolution of sibling cannibalism. This model suggests that the adaptive value of sibling cannibalism may be explained by the relationship between three main factors: (1) the level of relatedness between the victim and the cannibal; (2) the costs; and (3) benefits associated with cannibalistic behavior [3], [26], [27]. Cannibalistic behavior will be favored when the relative benefit to the cannibal exceeds the cost to the victim, weighted by its relatedness [27]. In addition, cost in inclusive fitness decreases if cannibals and victims are less kin-related [2], [25], [27]. Three important predictions arise from this theoretical framework: (1) sibling cannibalism will occur more frequently among individuals with low relatedness (i.e. reduced costs on inclusive fitness); (2) sibling cannibalism will arise with a higher likelihood in polyandrous species (i.e. increased likelihood that half-siblings rather than full-siblings interact in a brood); and (3) the cannibalism rate will increase at lower levels of intrabrood relatedness. The few experimental studies conducted so far, mostly on terrestrial species, support these predictions. For example, larvae of the paper wasp, *Polistes fuscatus*, tend to cannibalize more distantly related victims [28]. Similarly, individuals of the honeybee *Apis mellifera* and larvae of the tiger salamander *Ambystoma tigrinum* avoid cannibalizing close kin [8], [29].

Although sibling cannibalism has been observed in many marine species from different taxonomic groups, the predictions derived from the kin selection model have not been tested to explain this behavior in the marine realm. However, compared to terrestrial species, marine species show different evolutionary histories and are subject to different environmental limitations than those found in terrestrial systems. In this study, we evaluated the effect of relatedness on sibling cannibalism patterns in the marine gastropod *Crepidula coquimbensis* (Calyptraeidae). Fertilization in this species is internal and males transfer sperm during mating. Females encapsulate and brood their offspring for a period of about 40 days. *C. coquimbensis* is a direct developer, and juveniles hatch from the capsules at the end of the incubation time. Previous studies in *C. coquimbensis* have shown that cannibal embryos engulf their victims and store them in a ‘crop’ (*sensu* Véliz *et al.* 2003 [4]) for consumption during development. According to laboratory observations, between 10 and 50% of embryos reach the final developmental stage within the capsules. This species displays gregarious behavior with one female and two to six males (i.e. potential mates) sharing the same micro-habitat, i.e. an empty shell of another marine gastropod (e.g. genera *Tegula* and *Argobuccinum*) [30]. Within the micro-habitat, any male can fertilize the female and the female can store sperm from several reproductive events. *C. coquimbensis* thus offers several interesting features for testing for a relationship between kin-relationships and cannibalism: (1) females do not provide alternative sources of food to the offspring for development [4]; (2) oxygen conditions deteriorate during development, which can enhance competition for oxygen [31]; (3) cannibalism among siblings has been reported and (4) multiple paternity within capsules (i.e. co-existence of embryos fertilized by sperm from different males) has been previously documented in this species [32]. In addition, within the calyptraeid family, intracapsular sibling cannibalism appears to be associated with the direct development strategy [33]; therefore, the study of factors and the mechanism driving sibling cannibalistic behavior in *C. coquimbensis* would help to understand the evolution of developmental modes in this group.

By constructing artificial embryo aggregations characterized by different levels of relatedness, we examined the rate of cannibalism and the influence of this type of a behavior on the effective number of fathers within an offspring array. We used five hypervariable loci (microsatellites) described by Daguin *et al.* (2007) [34], and previously used for paternity analyses by Brante *et al.* (2011) [32], to perform paternity analyses on embryos at the start of their development (i.e. before cannibalism) and at the end of the development (i.e. embryos that survived cannibalism). We also determined the consequences of cannibalism on final embryo size, since larger size at hatching is expected to provide selective advantages to surviving embryos [35], [36], [37].

## Materials and Methods

### Ethics statement

We obtained permits to collect individuals of *C. coquimbensis* at the Management Exploitation Areas (MEA) for Benthic Resources of Puerto Aldea, from the artisanal fishermen union of Puerto Aldea. This field study did not involve endangered or protected species.

### Sample collection

Empty shells of marine snails hosting *C. coquimbensis* males and females were collected from Puerto Aldea (30°17′32′′ S, 71°36′30"W), Chile in January 2006, and transported to the Estación Costera de Investigaciones Marinas (ECIM) at Las Cruces, Chile. Each hosting shell was transported in an individual plastic bag filled with seawater to prevent the loss of individuals associated with each shell or movement of males between shells. In the laboratory, we labeled the female and all males belonging to each hosting shell (referred to as a ‘family’ hereafter). Five families in which a brooding female was found were used to perform all the experiments. One female and between three and five males were observed in each family (Table 1). These families were the same as those used by Brante *et al.* (2011)[32] to carry out paternity analyses and the embryos inside the capsules of the females were used for the experiments described below.

**Table 1:** Number of males contributing to broods and effective paternity index (see Materials and Methods) at the beginning (Pre-cannibalism) and the end of the experiments (Post-cannibalism) in the High Level of Relatedness (HLR) and Low Level of Relatedness (LLR) treatments.

		N_father_		Effective paternity	
		Pre-cannibalism	Post-cannibalism	Pre-cannibalism	Post-cannibalism
HLR	Mother 1	4.0	4.0	3.3	2.5
	Mother 2	3.0	2.0	2.6	1.4
	Mother 3	5.0	4.0	4.2	2.9
	Mother 4	5.0	5.0	4.5	2.9
	Mother 5	5.0	5.0	4.7	3.0
LLR	Mother(1+2+3+4+5)	21.4 (0.19)	7.0 (0.27)	18.5 (0.18)	3.1 (0.14)

For the LLR experiments, values are mean values (S.E.) over eight aggregations all composed of the same initial set of matrilines.

### Cannibalistic behavior

**Figure 1:**
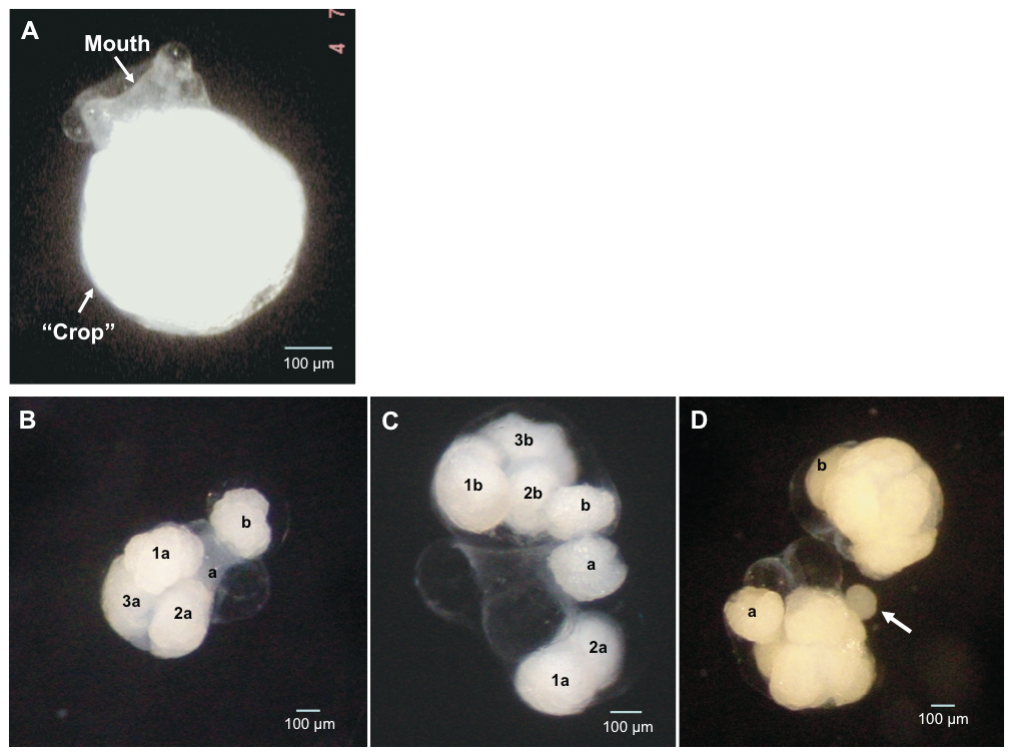
Sibling cannibalism in embryos of *Crepidula coquimbensis*. (A) Embryo of *C. coquimbensis* at the pre-veliger stage at which cannibalism occurs. At this stage, the elastic mouth can fully engulf the victim. All cannibalized embryos are stored in a “crop” and consumed [later] during embryonic development. Three different behaviors were observed: (B) Simple cannibalism by engulfing: one embryo (a) cannibalizes a smaller and less well-developed embryo (b). (C) ‘Hypercannibalism’ by engulfing: one cannibal (a) engulfs another embryo (b) which had previously cannibalized other embryos. The numbers inside the cannibal indicates previously cannibalized embryos. (D) ‘Hypercannibalism’ by sucking: one cannibal (a) cannibalizes another cannibal embryo (b) by sucking out its contents. The arrow indicates embryonic material extracted from embryo a by embryo b.

**Figure 2:**
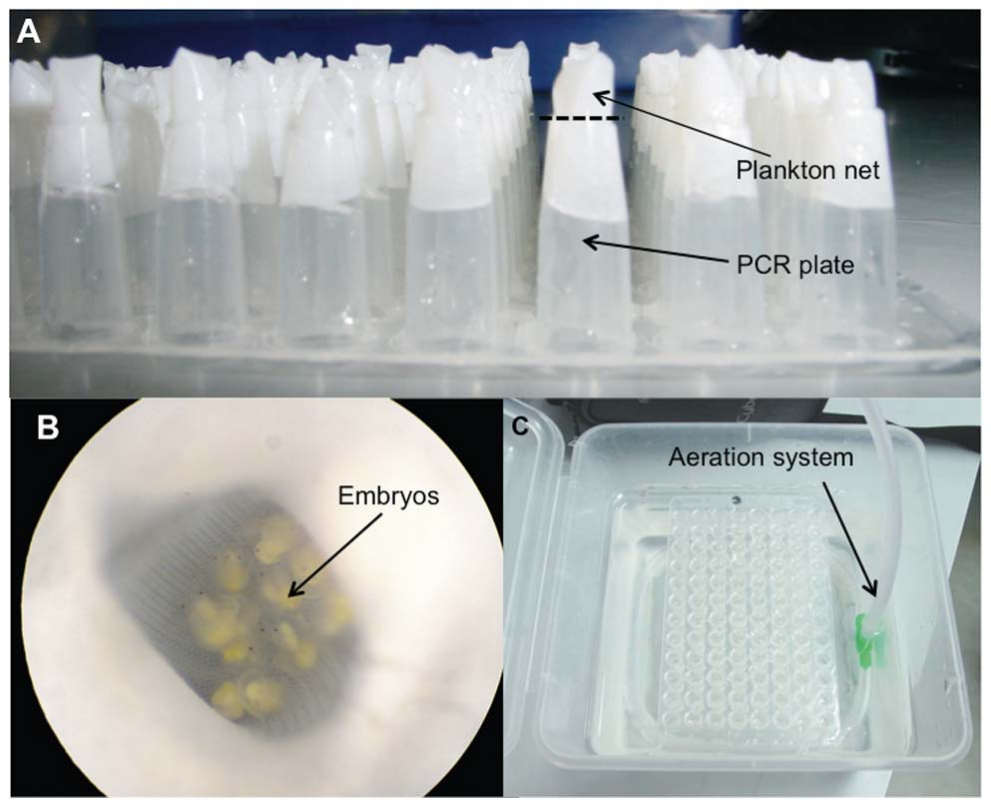
Artificial incubation chambers used to rear *Crepidula coquimbensis* embryos. (A) Phytoplankton netting was glued to the bottom of polypropylene 96-well V-bottom PCR plates whose ends had been cut off (segmented line). (B) Aggregations of embryos were maintained inside each well. (C) Plastic boxes filled with UV-sterilized filtered seawater, supplemented with antibiotics, were used to cultivate embryos in the customized PCR-plates. Water was continuously aerated by using perforated tubes connected to an air pumping system.

First, to describe intracapsular sibling cannibalism, we removed embryos at an early developmental stage, i.e. pre-veliger stage (embryo with a developed “crop” and no velum; Fig. 1A), from different capsules and cultivated them in artificial incubation chambers. These artificial chambers were made by cutting the ends off polypropylene 96-well V-bottom PCR plates (see ref. [38] and Fig. 2). Phytoplankton netting (45 µm) was then glued to the bottom of each well, thereby keeping individual embryos or groups of embryos separate yet exposed to the same experimental conditions (Fig. 2). The netting allowed for proper oxygen exchange between the water and the egg aggregation (see Brante *et al.* 2009 for methodological details) [38]. Antibiotics and UV-sterilized and filtered seawater (0.45 µm; 14°C) were used to prevent bacterial contamination. Water was continuously aerated and changed every other day. For 10 days, embryos were observed under the dissecting microscope and the various cannibalistic behaviors were recorded.

### Effect of relatedness on the intensity of sibling cannibalism

In *C. coquimbensis* fertilization is internal with direct copulation between males and sedentary females. Before fertilization, it is not possible to control the relatedness level, paternity identity and other factors (e.g. sperm storage, maternal effect, male quality). Consequently, it was not feasible to use a traditional experimental design to construct families and offspring arrays in *C. coquimbensis*. We thus evaluated the effect of intrabrood relatedness on the cannibalism rate by constructing artificial embryo aggregations mimicking different levels of relatedness (as measured by the relatedness coefficient *r*). We constructed 23 experimental embryo aggregations, using for each of them, 105 pre-veliger embryos sampled from the capsules of one to five brooding females. These 23 aggregations were used in three different treatments characterized by different mean sibling-relatedness levels according to the maternal origin of the 105 embryos: (1) 5 aggregations with a high level of relatedness (**HLR**); for each HLR aggregation, 105 embryos were randomly chosen from one capsule of a single female and reared together; thus, the five artificial aggregations each came from one of the five females; (2) 10 aggregations with a medium level of relatedness (**MLR**): 35 pre-veliger embryos were selected from one capsule from one female and this was done for three females; the resulting 105 embryos were pooled together for rearing; with five females, there was a total of 10 different combinations; and (3) 8 aggregations with a low level of relatedness (**LLR**); we pooled 21 pre-veliger embryos from one capsule of each of the five females to complete a total set of 105 embryos per aggregation; this was repeated eight times to get eight aggregations. Although some experimental aggregation treatments are not found in nature (siblings from different mothers), they were performed to exaggerate the cannibalistic responses with respect to relatedness level.

Mean relatedness level for each treatment (HLR, MLR and LLR) was estimated by genotyping embryos of each aggregation using five microsatellite markers (see below for genotyping protocol) and calculating pairwise genetic relatedness using Wang's triadic estimator implemented in the Coancestry 1.0.0.1 software package developed by Wang (2011) [39]. The average relatedness (considering all pairwise comparisons within aggregations) was calculated for each treatment. Because DNA extraction is destructive, it was not possible to run the genetic analyses at the start of the experiment on the exact same embryos used to make artificial aggregations, monitor cannibalism behavior and estimate paternity at the end of the development. Therefore, an alternative method was used to estimate relatedness level, multiple paternity and male participation at the beginning of the experiment (before cannibalism). Females of *C. coquimbensis* store and mix sperm of different males before fertilization. The relatedness of embryos within a given capsule is thus an accurate estimate of the relatedness of the other capsules and of the whole brood [32]. Therefore, for each treatment, the mean relatedness at the start of the experiment was estimated by using a genotypic dataset obtained from a previous paternity analysis [32] of other early embryos from the exact same broods (at least 56 embryos per brood were genotyped) and by constructing 100 simulated aggregations for each treatment with the same maternal origin and proportion of embryos as described for the experimental aggregations. Relatedness coefficients at the pre-veliger stage were computed for each of the 100 simulated datasets to check for consistency of the results. Comparison of relatedness between treatments (HLR, MLR and LLR) at the beginning of the experiment was performed with a one-way ANOVA after testing for normality and homogeneity of variance with Shapiro-Wilk and Brown-Forsythe tests, respectively. An *a posteriori* Tukey test was used when significant differences were detected.

The embryo aggregations were cultivated in artificial incubation chambers, and maintained in aerated UV-sterilized and filtered seawater (0.45 µm) for 50 days, time needed to complete development at the incubation temperature of 14°C. Antibiotics were added to prevent bacterial contamination and water was changed every other day. This protocol has been successfully used to cultivate embryos of *C. coquimbensis* and other calyptraeid species [38]. To quantify the effect of relatedness on cannibalistic responses at the end of the experiment (after cannibalism occurred), we estimated the cannibalism rate (number of cannibalized embryos compared to the initial number of embryos) and the final shell size of surviving individuals (juveniles) for each aggregation. Comparisons between treatments (HLR, MLR and LLR) were performed using separate one-way ANOVAs for both variables after testing for normality and homogeneity of variance with Shapiro-Wilk and Brown-Forsythe tests, respectively. When significant differences were detected *a posteriori* Tukey tests were performed.

### Effect of relatedness on victim choice and parental reproductive success

Using only the two extreme embryo aggregation treatments (HLR and LLR), we determined the effects of sibling cannibalism on parental contributions in *C. coquimbensis*. We compared the relatedness coefficient and paternity assignment before (i.e. pre-veliger stage) and after (i.e. at hatching) cannibalism using parentage analyses based on genotyping five microsatellite loci (see below for genotypic data and molecular methods). Computations of the relatedness coefficient at the start of the experiment were based on simulated aggregations as explained above. Relatedness level after cannibalism (i.e. at the end of the experiment) was estimated by genotyping between 52 and 74 juveniles per HLR aggregation and between 10 and 13 juveniles per LLR aggregation. The lower number of juveniles genotyped in the later treatment was due to the much lower number of surviving embryos arising from the high cannibalism rate observed (see Results). Pairwise genetic relatedness was calculated using Wang's triadic estimator implemented in Coancestry 1.0.0.1 [39] and the mean relatedness level calculated for each aggregation. Mean relatedness level before and after cannibalism was compared with Student *t*-tests for each treatment (LLR and HLR). Normality and homogeneity of variance was tested before analyses.

To determine if male reproductive success changes before and after cannibalism, we evaluated the percentage of contribution of the different males to the aggregation at the start (pre-veliger stage) and the end (juvenile stage) of the experiment. The changes were measured as the number of embryos assigned to a given father after cannibalism (at the end of the experiment) minus the number of embryos before cannibalism (at the start of the experiment) assigned to the same parent. The contribution of the different males at the beginning of the experiment (pre-cannibalism) was estimated from parentage analyses using genotypic dataset obtained with pre-veliger embryos as described above (see § ‘*Effect of relatedness on the intensity of sibling cannibalism*’). Parentage assignment across simulated aggregations was identical, and the parental profile (i.e. the percentage of assigned embryos per males and females) could be determined at the start of the experiment for each treatment. Male contributions after cannibalism was estimated from parentage analyses performed on juveniles at the end of the experiment (i.e. samples used to estimate relatedness level after cannibalism). The parentage analyses were performed using CERVUS 2.0 software [40] and following the protocol of Meagher (1986) [41], based on maximum likelihood calculations. All males sharing a hosting shell with brooding females were considered as candidate fathers. For the LLR aggregations, the parentage assignment also included the five females as eggs from each of them were used to construct the LLR experimental aggregations. Parentage was assigned to the individual with the highest log-likelihood ratio (LOD). To assess the statistical significance of the LOD score, computer simulations (10,000 iterations, based on allelic frequency of the entire population) were performed. Changes in the contribution of males to the brood (i.e. percentage of fatherhood) between the beginning and the end of the experiment were tested with chi-square tests. In addition, the effective paternity index (K_E_) was computed [42]. This index comes from an index used by community ecologists (Simpson's diversity index [43]). K_E_ is defined as follows: 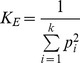
where *p_i_* is the proportion of offspring fathered by male *i* (*i* = 1…*k*, where *k* is the total number of fathers participating in the brood). K_E_ is maximum (i.e. equals *k*) when the relative contribution of the different fathers is equal (i.e. absence of skewed paternity).

For the LLR aggregations, we also tested if the final female representation departed from an even ratio (given that the same proportion of embryos from each of the five females was used as the initial condition) with a chi-square test.

### Genotypic data and molecular analyses

For relatedness and parentage assignment, genotypic data for adults and early-stage (pre-veliger) embryos were obtained at five microsatellite loci from a previous paternity study using the same ‘families’ [32]. For juveniles (i.e. after cannibalism), data were obtained following the protocols detailed in [32], [34]. Briefly, DNA extraction for adults was performed with a Nucleospin®Multi-96 Tissue Kit (Macherey-Nagel) following the manufacturer's protocol. For embryos and juveniles, the Higuchi protocol [44] was used for DNA extraction (see ref. 32 for more details). Samples were washed in PBS prior to DNA extraction to remove residual ethanol. Individual DNA was then genotyped at the same five microsatellite loci used previously [32], [34]: CcoqAC2F4, CcoqCT1H5, CcoqCT1F4, CcoqCT3F2 and CcoqCT1D10. Altogether, the five loci used were polymorphic enough to provide a paternity exclusion rate of 99% with a confidence level of 95%. Loci were amplified following the protocol described in ref. [34].

## Results

### Different cannibalistic behavior

Three different cannibalistic behaviors were observed between siblings: (1) cannibalism by engulfing (*sensu* Fioroni 1967) [45]: one embryo cannibalizes a smaller embryo by engulfing it (Fig. 1B); (2) hypercannibalism by engulfing: one cannibal engulfs another embryo which had previously cannibalized an embryo (Fig. 1C); and (3) hypercannibalism by sucking: one cannibal preys on another cannibal embryo, sucking its contents and killing it (Fig. 1D). In all cases, the victims are ingested by an elastic mouth and stored in the crop of the cannibal for [later] consumption during development (Fig. 1A). All victims showed embryonic segmentation and natatorium movements before cannibalism, suggesting that victims are eaten while still alive. In the case of the engulfing strategy, victims continued to move inside the crop of the cannibal embryo.

### Variation in the intensity and effect of the cannibalism with kin-relatedness

**Figure 3:**
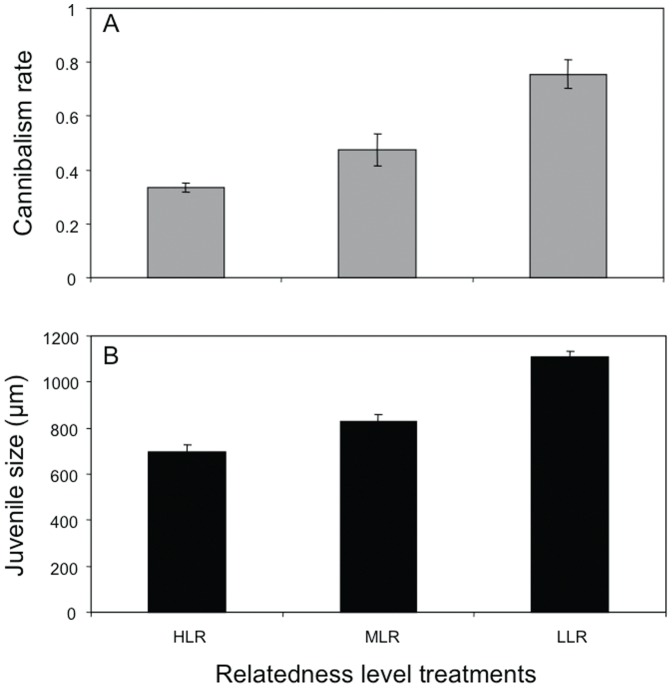
Cannibalistic behavior of *Crepidula coquimbensis* within the three categories of embryo aggregations: High (HLR), Medium (MLR) and Low (LLR) relatedness level. (A) Cannibalism rate and (B) juvenile size (i.e. after 50 days of development). Vertical lines correspond to SE.

The mean relatedness of the three treatments at the start of the experiment, estimated from simulated embryo aggregations, was 0.14, 0.18 and 0.27 for LLR, MLR and HLR treatments, respectively. The ANOVA and the *a posteriori* test showed significant differences between relatedness levels (ANOVA: F_2,20_ = 37.48, P<0.001; Tukey *a posteriori* test: in all cases P<0.05). By monitoring survival throughout development, we observed that the cannibalism rates varied from 0.28 to 0.84 among treatments, significantly increasing with decreasing values of relatedness (ANOVA: F_2,20_ = 22.44, P<0.001; Tukey *a posteriori test*: in all cases P<0.05; Fig. 3A). Mean hatching size of surviving embryos showed a similar pattern, increasing with decreasing relatedness levels (ANOVA: F_2,20_ = 28.25, P<0.001; Tukey *a posteriori* test: in all cases P<0.05; Fig. 3B).

### Change in relatedness and male reproductive success over time

**Figure 4:**
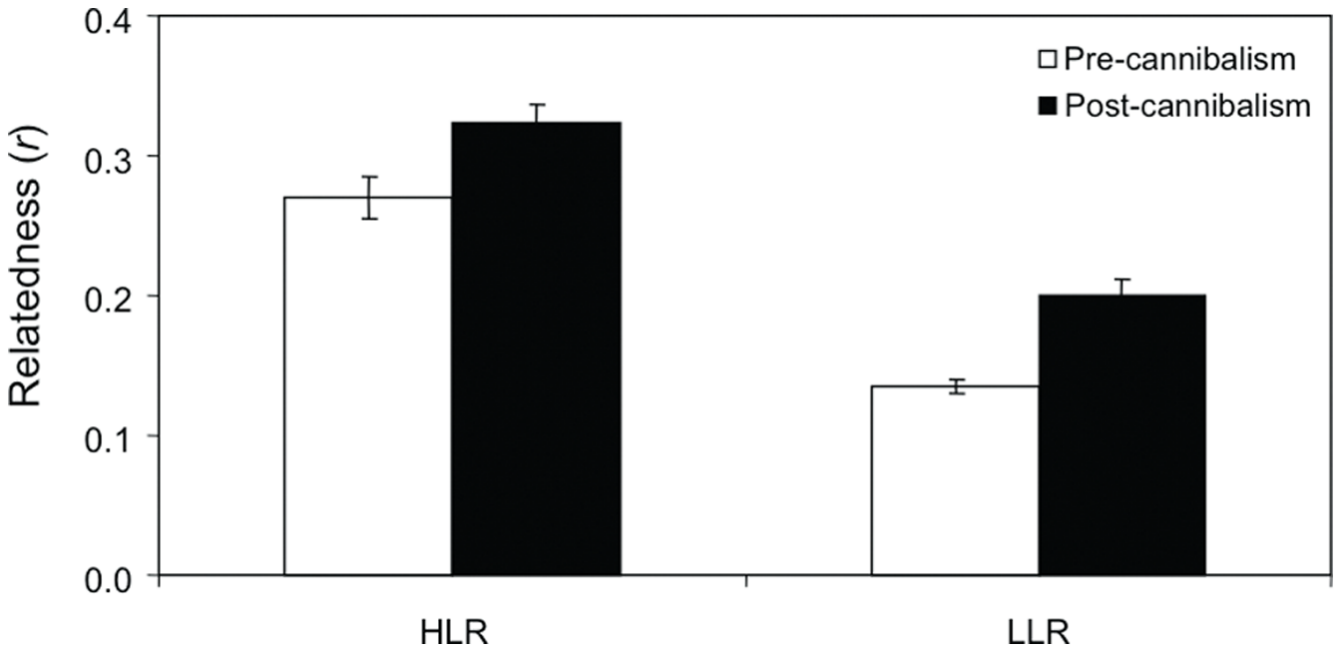
Relatedness coefficients (*r*) of the experimental embryo aggregations of *Crepidula coquimbensis*. Estimations of the mean *r* (± SE) in aggregations with high (HLR) and low (LLR) relatedness levels, before and after cannibalism.

Mean relatedness level within aggregations after cannibalism ranged from 0.32 to 0.20 for the HLR and LLR treatments, respectively. Student *t*-tests showed a significant increase in relatedness after cannibalism in both treatments (HLR: *t*-value = −2.65, P = 0.019; LLR: *t*-value = −4.90, P<0.001; Fig. 4).

**Figure 5:**
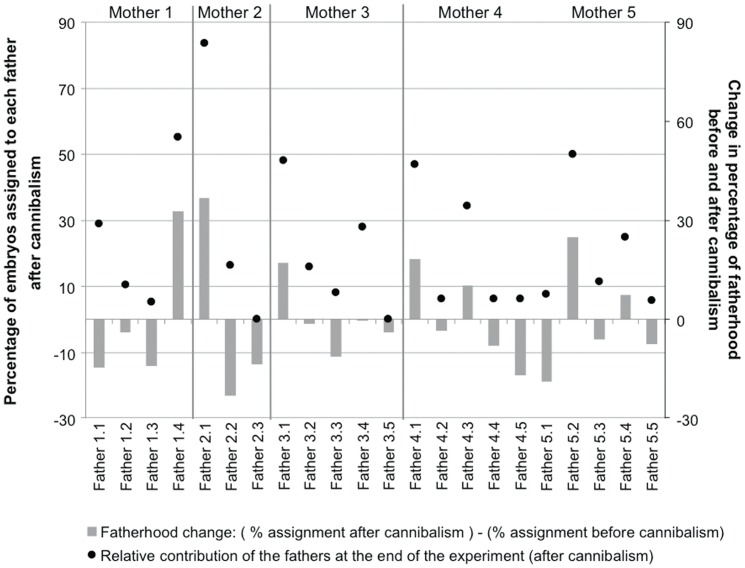
Cannibalism and paternal success within HLR aggregations during the development of *Crepidula coquimbensis* embryos. The percentage of embryos after cannibalism assigned to each father in the five HLR broods is indicated by black dots. Gray bars give the relative change in paternal contributions to the brood. The changes were measured as the number of embryos assigned to a given father after cannibalism (at the end of the experiment) minus the number of embryos before cannibalism (at the start of the experiment) assigned to the same father.

In the HLR treatment, the number of fathers changed only slightly with two to five males participating per capsule instead of three to five at the beginning (Table 1). Conversely, the relative contribution of each father did change significantly for all studied aggregations (female 1: χ^2^ = 65.9, df = 3, P<0.0001; female 2: χ^2^ = 4.1, df = 1, P = 0.1; female 3: χ^2^ = 21.9, df = 3, P = 0.0002; female 4: χ^2^ = 68.6, df = 4, P<0.0001; female 5: χ^2^ = 74.1, df = 4, P<0.0001; Fig. 5). The effective number of fathers strongly decreased after cannibalism compared to before cannibalism due to increased variance in male contribution over time (Table 1). In the HLR treatment, embryos that survived intracapsular cannibalism did not reflect the initial male fertilization success.

**Figure 6:**
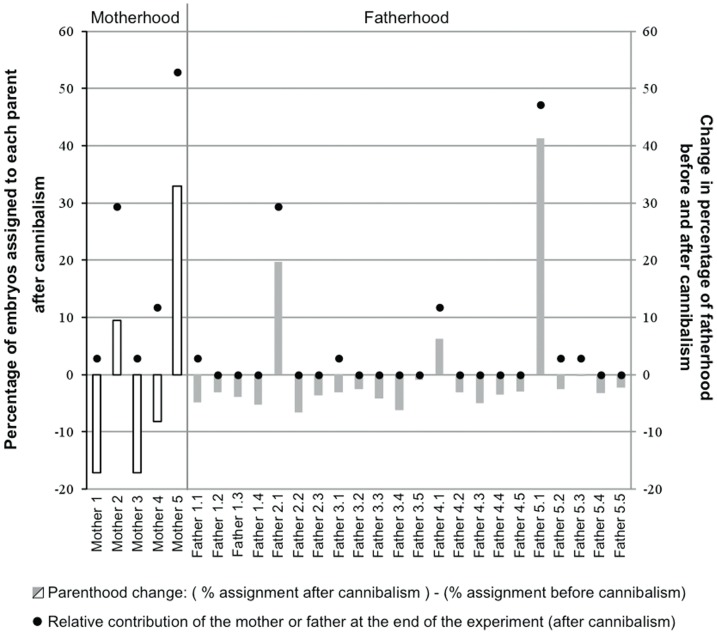
Cannibalism and parental success within LLR aggregations during the development of embryos of *Crepidula coquimbensis*. The percentage of embryos after cannibalism assigned to each parent in the LLR broods is indicated by black dots. In addition, the relative change in parental contributions to the brood is indicated by gray (males) and white (females) bars. The changes were measured as the number of embryos assigned to a given parent after cannibalism (at the end of the experiment) minus the number of embryos before cannibalism (at the start of the experiment) assigned to the same parent.

In the LLR treatment which combines embryos from each of the five females, embryos from females 2 and 5 showed the highest survival rates (Fig. 6). Regarding paternity profiles, as in the HLR treatment, the effective paternity was strongly reduced after cannibalism (i.e. from 18.5 at the beginning of the experiment to 3.1 after cannibalism; Table 1). In addition, significant differences in the relative contribution of the different males to the broods were detected over time (in all comparisons, chi-square analysis: P<0.05). Embryos resulting from the fertilization of female 2 by male 1, and of female 5 by male 1 exhibited the highest survival rates, representing 29.4% and 47.1%, respectively, of the embryos at the end of the experiment (see Fig. 6).

## Discussion

This is the first study examining the effect of relatedness on sibling cannibalistic behavior and its consequences on effective paternity in a marine invertebrate species. Our results showed that in *C. coquimbensis*: (1) three cannibalistic behavioral strategies were observed among embryos; (2) the intensity of sibling cannibalism was greater at the lower levels of relatedness, with important benefits in terms of size at hatching and (3) cannibalism between embryos was associated with an increased variance in male reproductive success. According to these results, we suggest that polyandry may play an important role in the evolution of sibling cannibalism in *C. coquimbensis* and that kin selection may effectively operate on early embryonic stages of this species.

Three distinguishable sibling cannibalistic behaviors were observed in embryos of *C. coquimbensis*: simple- and hyper-cannibalism by engulfing and hypercannibalism by sucking. Although the simple sibling cannibalism behavior has previously been documented in other marine gastropods [46], [47], [48], [49], this is the first report of intracapsular sibling hypercannibalism in a marine invertebrate. Intracapsular cannibalism has been observed in other species of the Calyptraidae group. For example, Collin (2003) [33] analyzed 78 species in this family and found that 10% of them displayed this kind of behavior. In all cases, these species have direct developmental strategies (absence of an intermediate larval stage). In addition, invertebrate species with encapsulation from other taxonomic groups show similar associations between sibling cannibalism behavior and direct development strategies, highlighting the importance of this behavior for understanding the evolution of developmental strategies in marine invertebrates. For example, the gastropods *Acanthina monodon* [24] and *Solenosteira macrospira* [49] from the Muricidae and Buccinidae families, respectively, and the polychaete worm *Boccardia proboscidea* (Spionidae) [50] show intracapsular sibling cannibalism and direct development. Under this scenario, the study of ecological, behavioral and genetic factors that modify the cannibalistic behavior in *C. coquimbensis* is clearly important for understanding not only the evolution of developmental strategies in the Calyptraidae family, but also the evolution of developmental strategies in the sea.

Under the kin selection model, it is expected that cannibalism between sibs will most likely arise in species showing a mating system that promotes multiple paternity [2], [3], [27], [51], as in species with multiple mating and/or sperm storage: both conditions increase the probability that more patrilines interact within a brood [27]. In the case of *C. coquimbensis*, a previous study demonstrated that females show a high level of multiple paternity, with roughly similar contribution of each male to the brood, by storing sperm for at least six months after copulation [32]. Kin selection also predicts that sibling cannibalism will increase at low levels of relatedness [3], [25], [27]. The few experimental studies reported in the literature support this prediction (for a review see Pfennig 1997) [3]. For *C. coquimbensis*, the cannibalism rate was highest for the treatment with the lowest relatedness level. Although we cannot estimate the pairwise relatedness coefficient between each cannibal and its victims to ensure that cannibals avoid full-sibs, the genetic relatedness analyses performed on eggs and surviving embryos shed light on the cannibalistic behavior of this species within the capsules. If cannibalism occurred randomly, no changes in the mean relatedness level of the aggregations would have been expected. Contrary to this expectation, the mean relatedness value of the artificial aggregations of *C. coquimbensis* significantly differed before and after cannibalism in treatments with high and low relatedness levels. In both cases, the relatedness increased towards the end of embryo development. These results suggest that some sub-groups of kin-related individuals are more efficient cannibals than other sub-groups of sibs. The fact that mean cannibalism rate increased at lower levels of relatedness and that the cannibalistic behavior does not occur at random within aggregations, strongly suggests that cannibals have the potential for kin recognition.

Additional evidence supporting the idea of non-random sibling cannibalism in *C. coquimbensis* comes from the parentage analyses performed on embryos before and after cannibalism. Results showed that embryos from some specific crosses (full-siblings) have a clear cannibalistic advantage and higher survival. For example, offspring from female 2/male 1, and female5/male 1 exhibited the highest survival rates in the LLR treatment. Although the experimental design does not allow us to separate the roles of the paternal and maternal factors in the higher survival rates of some embryos exposed to cannibalism, two lines of evidence suggest that father-derived traits may confer some advantages to their offspring. First, within a given aggregation, only one or two fathers dominated reproductive success at the end of the experiment. This over-representation was not explained by their fertilization success as the initial contribution of the fathers to the brood was roughly equal [32]. Second, one of the two most successful males at the end of the experiment in the LLR aggregations (i.e. father 2.1) was also successful within the HLR aggregation (see Fig. 5 and Fig. 6). However, the relative low success of embryos from father 5.1 in the HLR aggregations compared to their high success in the LLR treatment suggested more complex interactions. Based on the present study design, it is difficult to explain such contradictory observations. One hypothesis could be that cannibal abilities of embryos of *C. coquimbensis* could be influenced by an interaction between parental factors and physical conditions of the intracapsular environment. Dedicated experiments are needed to test for this hypothesis.

Size at hatching is positively correlated with the survival probability of early life stages in several marine gastropods [35], [36], [37]. In the present study, we showed that cannibalism may confer important benefits to cannibal embryos of *C. coquimbensis*: larger juvenile sizes were observed at higher cannibalism rates. The synergistic relationship between polyandry, encapsulation and cannibalism may also play an important role in the evolution of the direct development strategy in marine invertebrates. As mothers of *C. coquimbensis* do not provide food to their offspring during encapsulation, cannibalism is the only way for embryos to gain resources over a long period of time. Cannibalistic offspring grow and complete their entire embryonic development inside capsules. Avoiding the pelagic larval period reduces exposure to the high mortality risks associated with the first stages of a free-living state [52].

Many marine species from various taxonomic groups encapsulate their progeny during embryonic development [53], including gastropods, polychaetes, cephalopods, sharks, etc. Although encapsulation has been identified as an efficient mechanism for protecting offspring from different sources of mortality, particularly in variable and/or harsh environments [53], the capsule wall promotes competitive interactions between embryos by limiting resources [24], [54], [55]. For example, food and oxygen has been shown to be limited within *C. coquimbensis* capsules and can affect embryo survival and growth rates [38]. In *Acanthina monodon*, another gastropod species with similar intracapsular cannibalism behavior, low intracapsular oxygen availability may intensify cannibalism [24]. As indicated in the present study, cannibalism does not appear to be a random behavior. The co-occurrence of full- and half-siblings within a capsule may promote asymmetric competition between different paternal lineages, and ultimately determine differential male reproductive success. Therefore, the intracapsular environment may promote parent-sibling and sibling-sibling conflicts [49], [56]. Although family conflict has been largely studied in terrestrial species [27], [57], [58], similar studies are scarce in the marine realm (see [56]). The life history characteristics of *C. coquimbensis* make it an interesting biological model to test hypotheses related to family-conflict theory [57].

Finally, when considering only the HLR aggregations which mimicked natural conditions (i.e. embryos from one single brood), a striking increase in the variance in male reproductive success was observed after cannibalism as measured by the decrease in the effective paternity index. Cannibalism can thus also be considered as a driver of a reduced number of effective breeders in natural populations with subsequent consequences in terms of genetic drift and sweepstake effects [59], [60]. Therefore, intracapsular cannibalism in *C. coquimbensis* could have important consequences at ecological and evolutionary scales. Integrative experimental approaches, drawing on genetic, laboratory and field work, carried out in other taxonomic groups with similar strategies must be considered to identify more general patterns and understand the interplay between embryo behavior and evolution of life cycles in the sea.
